# Safe Game: Hygienic Habits in Self-Consumption of Game Meat in Eastern Spain

**DOI:** 10.3390/foods11030368

**Published:** 2022-01-27

**Authors:** Victor Lizana, Ana Muniesa, Jesús Cardells, Jordi López-Ramon, Jordi Aguiló-Gisbert, Juan M. Lomillos, Christian Gortázar

**Affiliations:** 1 Servicio de Análisis, Investigación, Gestión de Animales Silvestres (SAIGAS), Facultad de Veterinaria, Universidad Cardenal Herrera-CEU, CEU Universities, Alfara del Patriarca, PC46115 Valencia, Spain; jcardells@uchceu.es (J.C.); jordi.lopez1@uchceu.es (J.L.-R.); jordi.aguilo@uchceu.es (J.A.-G.); juan.lomillos@uchceu.es (J.M.L.); 2 Wildlife Ecology & Health Group (WE&H), Universitat Autònoma de Barcelona (UAB), PC08193 Bellaterra, Spain; 3 Departamento de Patología Animal, Área de Sanidad Animal, Facultad de Veterinaria, Universidad de Zaragoza, PC50013 Zaragoza, Spain; animuni@unizar.es; 4 SaBio Instituto de Investigación en Recursos Cinegéticos IREC (CSIC—UCLM—JCCM), PC13005 Ciudad Real, Spain; christian.gortazar@uclm.es

**Keywords:** cross-contamination, game meat, food hygiene, hygienic habits, waste management

## Abstract

We used anonymous questionnaires to assess the hygienic and sanitary aspects of game meat self-consumption in Eastern Spain as the first step towards a health risk assessment. The survey yielded 472 valid interviews from active hunters. The maximum possible score was 65 points (average 29 ± 8; range 1–52). Most participants were men (95%), but women achieved significantly better scores (*p* = 0.003). Hunters above 65 years old scored significantly lower results than younger groups (*p* = 0.007). The score increased with the educational level (*p* = 0.046). A 92% of the collaborators consumed game meat. Veterinary inspection and freezing were irregular among the participants. Most respondents declared carrying the animals in their personal vehicles. Of the dressing process, 61% of sites were outdoors, 68% of the participants declared using specific knives, 64% used the same clothes as in the field, and 42% used disposable gloves. The most usual way to dispose of the remains was garbage containers (41%); offal abandonment in the field was 33%, and 13% fed domestic animals using the remains. We conclude that public health authorities should increase their interest in the self-consumption of game meat. Clear guidelines about domestic dressing facilities and hygienic habits should be published, these being essential when looking for synergies with hunter associations.

## 1. Introduction

Game meat is a particularly appreciated product due to its high culinary [1] and nutritional value (high protein and mineral content and low fat and cholesterol levels) [2,3,4]. In developed countries, free game is perceived as an organic product [5,6] free of antibiotics and other pharmacological compounds [7]. Sustainable hunting is seen as a way to maintain biodiversity and ecosystems [8], being embraced even by the locavore movement [9]. Rates of consumption are lower than those of domestic species, sometimes related to the difficulties in obtaining it [10]. In Spain, meat from wild game species represents only 2% of the total meat intake [11], and 59.6% of game meat is consumed by the hunters themselves or the hunters’ inner circle (relatives, friends, and neighbors) [12].

Due to its origin and special processing circumstances, game meat faces its own challenges and may represent a higher sanitary risk in comparison with farm-origin meat. Wildlife does not have the sanitary status achieved by farm production; the animals are culled in the field [13,14] and, especially in domestic consumption, carcasses are often eviscerated and skinned under poor hygienic conditions [14,15,16]. Some issues such as carcass contamination, promoted by bullet trajectory [13,17,18], heavy metals from ammunition remains [19,20,21,22], or the time between death and skinning and dressing [13,23], are specific concerns in game meat use.

When animals are correctly shot and dressed, microbiological scores of fresh carcasses may be low, similar to those from domestic species slaughtered in controlled conditions [6,13,17,24,25]. Notwithstanding this, levels of biotic contamination can be highly variable due to the shifting conditions in which wild game is killed and processed in the field and their carcasses transported, handled, chilled, and stored [13,14,16,17,26]. External factors, such as warm/cold seasons, contribute to microbiological growth as well, being difficult to mitigate [27].

Zoonotic viruses, bacteria, and parasites are commonly found in game carcasses. Health hazards will vary depending on the animal species, the degree of fecal contamination, the time elapsed from death to butchering, and the conditions of dressing and cooling [28,29,30,31,32,33,34,35].

Opinion polls are a common tool to obtain information from stakeholders about many topics. Fish and wildlife management professionals use public opinion and attitude surveys to facilitate an understanding of their constituents [36]. Those surveys have been used to consult attitudes towards hunting and fishing [37,38,39], game meat consumption [38,40], or the opinion about governmental wildlife agencies [41]. Public health authorities also use this methodology to obtain information about the food hygiene practices of their citizens [42,43].

We used opinion polls to assess the hygienic–sanitary aspects of game meat processing and self-consumption in the Valencian community, Spain, as a first step to obtain a human and environmental health risk assessment.

## 2. Materials and Methods

### 2.1. Study Area

**Figure 1:**
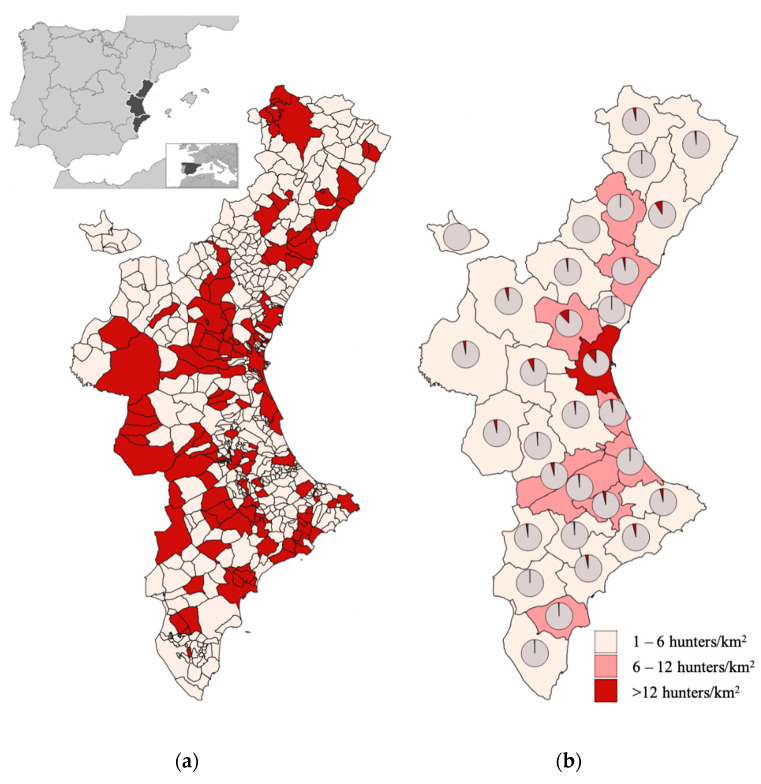
Survey distribution throughout the study area: (**a**) surveyed municipalities; (**b**) survey proportion per county and hunter density.

The present study was conducted in the Valencian community (Eastern Spain) (see Figure 1), a 23,255 km^2^ autonomous region with 4.9 million inhabitants [44]. The region is divided into 31 districts and 542 municipalities, with four cities over 150,000 inhabitants (Valencia, 786,000; Alicante, 335,000; Elche, 234,000; Castellon, 167,500) and another 24 smaller ones between 30,000 and 85,000. Most of the population is located on the Mediterranean coastland; meanwhile, the inland population density is low.

The target of the survey was hunters, estimated at 36,829 [45]. Although hunting is not allowed in the whole territory (it is forbidden in protected areas, densely populated districts, and buffer zones surrounding infrastructure and isolated buildings), 19,033 km^2^ (82% of the total) is enabled for this activity [46]. Except for wild waterfowl, which are more common in coastal wetlands, most animals are hunted in the inland counties with 90% woodland areas [46].

In the Valencian community, veterinary inspection is mandatory for those hunting events that plan to sell the meat. The carcasses generated should be primarily inspected by a veterinarian in the field, then transported to game slaughterhouses in neighboring regions (because there are no specific slaughterhouses in the territory), where a second veterinary inspection will take place. Under the Regional Decree 201/2017 [47], individual hunters can sell small amounts of game meat to the final consumer or to a commercial establishment selling directly to the final consumer, but the requirements (e.g., authorized veterinarians, local slaughterhouses focused on game meat, forms, taxes) have not been completely implemented yet. Hunters do not have an obligation to pass a veterinary inspection of the carcasses (except for *Trichinella* spp. in wild boar) if the intention is self-consumption.

### 2.2. Opinion Poll

Structure: The survey forms were composed of 35 questions (see Appendix A). Some of them admit multiple-choice answers (e.g., species harvested, hunting modalities), while others are mutually exclusive (such as gender or level of studies). Four different content blocks form the questionnaire:

1. Consent to participate: Prior to the main questionnaire, potential responders had to agree by marking a specific box and were informed about the voluntariness, topic, and goal of the survey and about the aim of publishing the results at the end of the study. They were also informed that their data would be treated anonymously.
2. Generalities: Questions about personal information (gender, age, level of education, profession, place of residence) and hunting activity (animal species, modalities, location).
3. Game meat consumption: Species consumed, amount of meat, culinary preparation.
4. Dressing and butchering: Veterinary inspection, game transport conditions, facilities and tools used, protective clothes and equipment, cleaning processes, and waste disposal.

The third block was established and inspired by the model of structural, hygienic, and transport requirements in game meat processing used by Vinhas [48] and Vieira-Pinto [15].

Panel of experts: The first draft was sent to a selected group of researchers and public officers, chosen because of their relevance and knowledge in food hygiene, wildlife management, zoonoses, public health, and hunting practices. After several meetings and corrections, a fourth version of the questionnaire was considered appropriate to the survey goals.

Validation: Once the consensus draft was ready, it was given to several trusted hunters to evaluate the comprehension level of the question pool to detect if any information considered relevant by the hunters was missed. The survey was validated with minor modifications regarding the meaning of some abbreviations, which were modified in the final version.

Survey: Hunters were randomly selected to achieve regional representativeness. A physical copy of the questionnaire was given to the collaborators. However, no copies were offered to those answering on the internet, as we tried to avoid non-desirable behaviors such as poll crashing [36] or undesired responses from hunters belonging to other regions. To better comply with the obligations mentioned in Regulation (EU) 2016/679 on personal data protection, the form was anonymous. In addition, we consider that an anonymous questionnaire will achieve a higher level of truthful answers

### 2.3. Statistical Analysis

To evaluate potential differences in social–educational background and hygienic practices, a scoring system was established using the model created by Vieira-Pinto [15] as a starting point. This score values the hygienic procedures, facilities, supplies, and waste disposal of the interviewed hunters. The distribution of each variable was studied with the Kolmogorov–Smirnov test. Non-parametric tests were used for those parameters with a non-normal distribution (*p* < 0.05), using the Mann–Whitney U-test when comparing among two categories and the Kruskal–Wallis test to compare more than two categories.

The minimal sample size was established in 381 polls for a survey based on 36,829 hunting licenses for a 95% confidence level and 5% precision with EPIDAT v.4.2 software [49].

## 3. Results

The survey period began in January 2018 and ended in February 2020. A total of 516 questionnaires were filled out, but 44 (8.52%) were rejected due to missing answers or because the respondents lived out of the surveyed area. The final amount of 472 valid interviews was considered enough for the targeted population. Questionnaires from 128 municipalities were held, representing 23.8% of the total number of municipalities into which the territory is divided (Figure 1).

The maximum possible score, corresponding with optimal hygienic behavior, is 65 points. The average score achieved by the surveyed hunters was 29 points (highest 52, lowest 1) with a deviation of 8 points (median and interquartile range, because data distribution, studied through the Kolmogorov–Smirnov test, is not normal, *p* = 0.030). An overwhelming majority of participants in our survey were men (95.1%), but women achieved the best scores (*p* = 0.003). Among ages, 18.8% of the participants were between 18–30, 44.4% 31–50, 27.5% 51–65, and 9.3% >65 years old. Age-related differences in the hygiene score were marginally significant (*p* = 0.053). However, when comparing the results from >65 y.o. hunters with those from the rest of the interviews, the result was *p* = 0.007. Referring to educational background, 6.1% had no studies, 37.8% had primary education, 39.3% had secondary–professional education, and 16.6% had university degrees. The total score value increased significantly (*p* = 0.046) as the educational levels got higher.

The largest consumed big game species in our survey was wild boar (*Sus scrofa*) (22.5%). Among small game, wild rabbit (*Oryctolagus cuniculus*) (24.4%) and red-legged partridge (*Alectoris rufa*) (20.7%) are the most popular ones. The preferred hunting modalities (90.6%) involve hunting dogs or other assistant animals (ferrets or raptors). In total, 92.1% of the collaborators consume the hunted prey; 40% of them allocate game meat to their own families, 9.4% give the meat to relatives, friends, or neighbors, and 48.4% to both previous assumptions. Only 2.1% of the surveyed hunters declared selling the meat. Most hunters (59.3%) cooked it for consumption. Preparations such as raw sausages or salt-cured meat are less common (16% and 10.1%, respectively). Pickled preparation (13.7%) is also common, especially among small game hunters, while other options are scarce (smoked (0.4%), pâté (0.1%), or raw (0.3%)). Among the 7.9% who do not consume their game meat, the main situation was the lack of time for meat dressing (69.5%), rejection (19.5%), lack of culinary interest (8.7%), or health problems (2.17% with uric acid rising).

Sanitary behaviors such as veterinary inspection or freezing were irregular among the participants. Only 22.4% always resort to a veterinarian, while 11.9% do it “usually” and 13.4% “not normally”. Most results (34%) are for those who approach a veterinarian only for *Trichinella* diagnosis after hunting wild boar, while 18.2% (essentially small game hunters) admitted to never consulting a veterinarian. The need to freeze game meat before consumption is felt as mandatory for some hunters; 40.6% declared freezing it “always” while 48.2% do it “usually”. A lower 7.6% admitted putting the meat in the freezer “occasionally” and only 3.5% “never” do it. Those hunters who declared freezing game meat were additionally asked about the period they let the meat freeze. Of these, 17.3% freeze the meat for less than 1 month, 39.1% between 1 to 2 months, 34.5% between 2 to 6 months, and 9.1% more than 6 months.

Focusing on cross-contaminations, several questions were asked about the possible weaknesses in meat processing, personal hygiene, cleaning protocols, or suitability of dressing facilities. About hunted-animal transport, most respondents (74.4%) declared carrying the animals in their personal vehicles; 13.1% prefer to move them in the back of an open pickup or open trailer; 8.5% transported the carcasses in the back of a pickup with a canopy or an enclosed trailer. The best option from a food safety point of view, an exclusive vehicle or trailer with a refrigeration system, was available for only 3.6% of the surveyed hunters. Only 3.2% of the interviewed hunters had authorization to transport animal by-products (ABP) not intended for human consumption. In fact, except for those involved in farming, the meat industry, or animal health, this terminology was unknown. About evisceration, 19.7% of the interviewed hunters declared dressing the prey immediately in the field. For 28.7%, the time elapsed was less than 2 h; 2 to 4 h for 27.7%, 4 to 6 h in 13.5%, 6 to 8 h in 4.1%, and 5.1% exceed over 8 h; 81.8% of respondents owned their own dressing facilities, 7% performed the process in another person’s place, 7.8% in a collective property (hunting clubs), 1.5% were done in public places (commonly obsolete municipal slaughterhouses granted by local authorities), and 1.9% of the answers indicated dressing the animals directly at the killing site. Of the dressing sites, 60.8% were outdoors (killing site, yards, or patios), while 39.3% were indoor installations. Only 32.3% of the facilities were specifically dedicated for game dressing, while the rest had shared uses with other activities (yard 19.3%, storage 15.7%, garage 15.5%, workshop 1.7%) or even with activities, allowing the contamination of food and meals or human contagion (pantry 5.7%, kitchen 5.3%, meeting place 3.2%). Of the hunters, 91.6% had access to safe water in their dressing facilities, but only 54.8% (mainly those who dress the animals in their own homes) had hot water. Of the facilities, 5.9% had water not coming from supply systems but from irrigation and rainwater tanks. In 2.5% of the situations, the facilities had no access to water at all. Regarding carcass position when dressing and cutting tool utilization, 53.8% of hunters declared suspending the animals with a hook or a similar device, 36.4% eviscerated the animals on a table or bench, 4.9% displayed the bodies on the ground over plastic sheeting, 3.4% on a pavement floor, and 1.5% directly on a dirt floor; 68.1% of the participants declared using a specific set of knives for dressing, while the remaining 31.9% admitted using the same knives for other purposes. Some questions were made regarding the light sources in the dressing facilities. Of the responders, 22.4% depended completely on sunlight, while the rest had access to artificial sources. Incandescent bulbs were the most common devices (34.9%), followed by LED lights (14.8%) and halogen tubes (14.6%), while 9.5% used a combination of natural and artificial sources and a marginal 3.8% used portable manual devices (such as torches and headlamps). Given the potential for insects and rodents to contaminate food [50], the interviewed hunters were asked about the protective measures available in their dressing facilities. Regarding insects, the most common devices were insect gauzes in windows (34.5%), adhesive traps (8.7%), light traps (8.5%), and insecticide sprays (4.4%). Even so, 43.8% of the responders did not use any device to control insects. Concerning rodents, spring traps were the preferred devices (14.1%), followed by poison pellets (12.7%), glue traps (5.6%), cage traps (5%), and, finally, cats (0.8%). Most of the hunters referred to not using pest control strategies (61.8%). About cleaning protocols, 35.3% of users informed hosing down their facilities after dressing, while 24.5% prefer to mop the floor. The combination of both (hosing down and mopping) was also popular (14.9%). The use of scouring pads (9.3%) was referred when cleaning sinks, tables, and other surfaces. Dry brushing was the option for only 1.5% of hunters. Combinations of several of the previous procedures were reported in 10.5% of the answers, and 3.9% did not clean the dressing site in any way. About disinfectant use, the most common ones were bleach (27.4%), soap (8.4%), and floor cleaners (4.8%). Other options were marginal (ammonia 0.2%, caustic soda 0.4%). Almost half of the interviewed subjects (47.9%) do not use any kind of substance but water. Regarding the clothes they used during the dressing. 63.7% use the same clothes as in the field and 15.5% use covering clothes (such as a lab coat or a coverall) over the usual wear; 16.4% changed all their clothes to use specific clothing and only 4.4% use specific footwear (rubber boots). To evaluate the risk of suffering cuts, stabs, spills, or inhalations, a question was made about the use of personal protective equipment (PPE). The most common items were disposable gloves (42.1%), while other equipment was used by less than 5% of participants (leather gloves 4.6%, steel mesh gloves 4%, facial mask 2%, face shield 1.8%). Some hunters (5.5%) used more than one item simultaneously, disposable gloves and steel mesh gloves being the most common combination (1.7%). Most answers (44.9%) confessed to not using protective equipment at all. About personal hygiene, they were asked about the possibility of washing their own hands during and after meat processing in the slaughter facility; 45.4% declared having access to running water, 8.9% had access to water, but from an uncertain origin (e.g., rainwater, water well), and 2.3% had no option to wash their hands. In addition, 36.9% used soap and 6.4% hand sanitizers or disinfectants.

Regarding waste management, the most usual way to dispose of the remains was municipal garbage containers (41.3%). In 33.2% of cases, the answers showed the abandonment of offal and other remains directly in the field (11.1% were buried, 11.5% were left in an open area, and 10.6% were hidden in a pit cave or gully). Of the interviewed hunters, 12.8% declared to use offal to feed domestic animals (dogs 12.1%, cats 0.34%, pigs 0.34%). In 7.9% of the answers, the organic remains were disposed of in carcass containers (associated with the farming industry); 4.4% of users declared leaving the offal in vulture feeding stations, and a marginal 0.34% use incineration to dispose of the remains. Wastewater was eliminated through collective water networks by most hunters (53.5%); 30.2% released the contaminated water into rivers, ravines, or crop fields, 7.8% did not know the destination, 7.6% had access to a specific organic residue water treatment plant, and 0.8% stored the wastewater in septic tanks.

## 4. Discussion

This survey offers a wide spectrum view towards private game meat self-consumption, a scarcely studied topic concerning both wildlife management and public health perspectives. Although some key aspects cannot be evaluated by using opinion polls, such as the efficiency of dressing, hygiene and cleaning protocols, the time span needed to reach refrigeration/freezing temperatures, or the microbiological status, it still constitutes a useful tool for studying the social aspects implied, and it allows us to detect deep-seated procedural errors, training needs, and deficiencies in the facilities.

Despite the openness of game activity towards women in recent decades, it continues to be a mainly masculine practice [51,52], and only 1.15% of hunters from our study area are women [53]. This fact explains why only 4.9% of the surveyed people were huntresses. The marks achieved by women in our study were significantly higher than those of men. Several previous surveys have demonstrated a greater efficiency and dedication from women towards hygienic procedures [43] and a bigger concern about potential food safety risks and safer practices than men [42,54,55]. Our survey only detected marginally significant differences among age groups when compared altogether. However, in accordance with previous research [56], which has found more food safety malpractices in older adults, we compared the score achieved by the >65 y.o. group with the rest of the participants, finding a significantly lower total score in the oldest group. Consistent with previous studies, the level of education is a relevant factor in the final achieved score. People with very little or no educational background have a low understanding of food safety issues [57]. A higher level of formal education creates awareness in ensuring safer and more hygienic practices [58,59]. A higher awareness of potential risks is reached by people with university studies, with increased knowledge among those from health-related fields [55,60]. Experience can play an important role too, and it has been shown that training leads to better results in microbiologic contamination in game meat processing [25].

In reference to hunted species, our results are consistent with the official game statistics, showing that the most consumed game is also the most abundant one. Wild rabbit is the most hunted and consumed small game species (430,931 individuals—24.4% answers), red-legged partridges for birds (89,049—20.7%), and wild boar for big game (26,832—22.5%) [61]. In Spain, most authorized hunting modalities involve the utilization of assistant animals. Ferrets, raptors, and, especially, different dog breeds are used to help the hunters track, chase, and catch wild prey. Bite-borne microbiological contamination is a collateral effect of this utilization and a concern to food hygiene goals [62]. Unlike other parts of the country, the Valencia region does not have a culinary tradition in preparing sausages or cured game meat. This cultural behavior works as a self-protective strategy against some pathogens (such as the hepatitis E virus, toxoplasmosis, or trichinellosis) that can be transmitted by raw and undercooked meat [31,63,64,65]. Specific concerns between wild boar meat consumption and *Trichinella* spp. transmission were expressed by some hunters. Different strategies (or combinations of several procedures) are commonly carried out to reduce the risk of transmission, such as cooking the meat, freezing the carcasses for at least 2 months, and veterinary checking for *Trichinella* larvae.

Geographic locations where the animals have been shot can be notably far from the dressing facilities. Thus, transport conditions can be challenging to assure meat safety. The safer option (i.e., an exclusive vehicle with refrigeration) was selected by only 3.6% of the surveyed hunters, thus evidencing the lack of proper transport systems, comprising a potential risk for meat contamination. Furthermore, the most popular option (i.e., personal vehicles) may represent a supplementary public health risk due to the difficulties of cleaning up fluid spills, the utilization of the vehicle for private purposes, and the contamination of personal items sharing the space. European regulations show some legal uncertainties regarding the scope of the rules on ABP from wild game. Regulation (EC) No 853/2004 admits that, if good hunting practices are observed, intestines and other body parts of healthy wild game hunted in their natural habitat may be disposed of on-site. To ensure the health status of the animal, a new figure of “trained hunter” was created, understood as a person with additional training, able to make an on-site evaluation of animal health before veterinary inspection [66]. The Valencia Community Regional Order 3/2019 excluded this possibility, declaring graduates in veterinary medicine as the only professionals able to check the sanitary status of game animals [67]. Regulation (EC) No 1069/2009 considers the wild animals when suspected of being infected with diseases transmissible to humans or animals as Category 1, while the carcasses and body parts of healthy game animals which are fit for human consumption but not intended for commercial sale are classified as Category 3 [68]. All those regulations are focused on game meat commercialization, while self-consumption is not mentioned and could be considered a loophole and a threat to human health. The low rate (3.2%) of hunters with the authorization to transport ABP shows an extensive lack of knowledge about this regulation. The time elapsed between the death of the animal and the evisceration can be highly variable, depending on different factors such as the distance from the field to the dressing facilities or the hunting modality (it is longer in collective hunting, where participants are not allowed to leave their positions until the end of the hunt due to safety reasons [69]); 22.7% of hunters declared exceeding the time span considered to be critical for limiting bacterial spread from unbroken guts [70]. If fecal contamination is present because of abdominal shoots, the combination with high carcass temperatures for several hours presents a potential risk for human health. In fact, the contamination of game carcasses by feces and subsequent invasion by enteric pathogenic bacteria is rather common in game because, in general, animals are eviscerated and skinned under insufficient hygienic conditions [15,70,71]. Butchering and dressing animals are potentially risky processes in which the handler comes into contact with organic material and body fluids, potentially carrying disease-producing microorganisms [72] that can be transferred to surfaces and tools while handling meat [73]. There is an evident need for training in hunter communities about personal hygiene, protective measures, and the use of specific protective gear during game meat processing. The lack of use of these protective supplies has been demonstrated as a risk factor to people involved in dead animal handling [74,75,76]. Due to their high exposure to wild animals, hunters and butchers of wild game (among other collectives) are commonly exposed to animal pathogens, being the origin of contagion to other people. Public health monitoring programs looking after wildlife diseases should focus on those groups [77,78]. Lighting is a relevant factor for proper animal dressing in order to detect alterations that lead to discarding affected areas and performing the operations in a safe way. Nearly a quarter of the surveyed users (normally associated with outdoor facilities) depended totally on sunlight, which should be a matter of concern. Additionally, there is room for improvement in those facilities using incandescent bulbs, which should be properly enclosed to prevent glass contamination in meat in case of breakage [79]. Taking as a reference the European standard UNE 12464-1:2011 about the lighting of workplaces, the indoor dressing facilities must reach a 500 LUX light level [80], an unknown factor for self-consumers. There is room for improvement in dressing facilities because most of them are located outdoors; the facilities are then exposed to contamination by insects and dust [48] and are also used for other activities, in close contact with food and people. Insects and rodents are the origin or vehicle of microorganisms that are able to contaminate food and processing sites, being able to transmit *Salmonella* spp. [81,82], *Campylobacter* spp. [83], *Yersinia* spp. [84], and *Cryptosporidium parvum* [85], among others. For this reason, it is considered essential to implement pest control protocols, not only by access prevention or direct persecution but also by ensuring that control methods (traps, poison, cats) do not constitute a significant health hazard in themselves. Under proper maintenance and control, some measures are considered desirable, such as insect gauzes, insect light traps, or rat poison, while others should be discarded due to their implicit risk of becoming a source of contamination. Most common insecticide sprays used in domestic dressing facilities are not focused on the food industry, so the chemicals could remain on surfaces where raw food materials are displayed because of the absence of further studies about suspension periods and a lack of proper training in the users. This situation constitutes a potential source of toxic exposure through food intake [86]. Adhesive traps could be useful for early insect presence detection [87] but should not be used as a device to control pest populations. On a general basis, all animal access to dressing facilities must be avoided, thus impeding the entry of domestic cats and disqualifying felines as valid rodent controllers [88]. The quality of cleaning water access and lack of heating devices should be matters of concern. Surface and tool cleaning with hot water is a common method to reduce the pathogenic bacteria related to meat processing [89,90]. However, of the 91.5% of respondents who declared having water in their dressing facilities, only 54.8% had hot water and this does not imply reaching temperatures high enough to reduce bacterial presence in an effective way. The common practice of cleaning the carcass during dressing increases some bacterial counts [25]. Future actions should be taken to train the target population, aiming at domestic infrastructure improvement. Inadequate decontamination procedures applied to potentially contaminated utensils may present further risks of microbial contamination in subsequent meal preparations, thereby reducing the shelf life and product safety [56,91,92]. Almost one-third of the surveyed hunters declared using the dressing knives in other activities. The position of the carcasses when dressing can create additional contamination of the derived meat. Most surveyed hunters (90.2%) displayed behaviors considered appropriate, hanging up the carcasses (53.8%) [48] or putting them on a specific table or bench (36.4%). Animal dressing on the floor (on a plastic sheeting (4.9%) or pavement surface (3.4%)) can be considered minor options, with the worst-case scenario (evisceration on a dirt floor) [15] as anecdotical (1.5%). Cleaning protocols are key aspects of hygienic meat processing. It is essential to analyze the procedures to detect weaknesses that may signify a cross-contamination risk from inadequately sanitized facilities, health hazards due to bacterial proliferation, or the apparition of persistent bacteria [93]. Floor cleaning through hosing down or mopping may be seen as correct practices, but this can generate droplets and therefore should be discouraged during and immediately prior to production periods [94]. Using only water (the case of almost half of the interviewed users) would be useful only if high pressure is used. A proper combination of kinetic energy through the impact of water droplets, time, and temperature is enough to remove biofilms [95]. However, if those conditions are not achieved, then protein and fatty organic residues will remain, allowing the persistence of microorganisms. Bleach and its derivates are some of the most effective home sanitizers available, and they are also used in the meat industry [96,97]. Thus, its popularity in being used alone or combined with other disinfectants is evaluated as desirable. As commented previously about insecticides, disinfectants used for cleaning surfaces or tools in contact with food should be authorized for the food industry to guarantee the safety of the derived products.

Surfactants present in soap, detergents, and floor cleaners enable the removal of fatty organic remains and affect the cellular membranes of the microorganisms [98]. The combined use with a sanitizer such as bleach is the best practice. Inappropriate practices, such as dry brushing, which often results in the generation of bioaerosols, transferring floor microorganisms to food preparation surfaces [99,100]; the total absence of cleaning interest is a minor point in this survey.

Waste management is one of the main problems detected in the survey. Trash containers were the most common way to dispose of solid residues derived from game dressing and butchering (41.3%), but those structures are accessible to synanthropic mammal species such as brown rats (*Rattus norvegicus*) [101] or red foxes (*Vulpes vulpes*) [102], who can feed on the remains, thus being a source of disease spreading. It is known that free-living cats (*Felis catus*) tend to visit rubbish bins regularly [103], closing the life cycle of toxoplasmosis. Environmental contamination with oocysts of *Toxoplasma gondii* has been found surrounding rubbish deposits associated with feral cats [104]. Toxoplasmosis infection rates in local wild boar (the most killed big game) is 14.1% [105]; hence, organic remains are potentially risky; 33.2% of answers disclosed the abandonment of offal and other remains directly in the field. This method, being legal in some EU states (Regulation (EC) No 853/2004), is a source of contamination and a way to help parasites complete their life cycles [15,106,107,108]. Three species of *Trichinella* have been found in Spain (*T. spiralis, T. britovi,* and *T. pseudospiralis*) [109]. Under proper climatic conditions, infective *Trichinella* larvae can remain viable in carrion for a long period [110,111], acting as a source of infection for mammal scavengers [33] and synanthropic carnivores [112]; 12.8% of surveyed hunters use the remains to feed domestic animals (essentially dogs but also cats and pigs). Wildlife is a reservoir of a plethora of pathogens that is able to affect domestic species, for example, *T. gondii* [105], Aujeszky’s virus [113], *Alaria alata* [114,115], *Echinococcus granulosus*, transmitted from ruminants’ offal [116], and *E. ortleppi* from wild boar viscera, accessible to dogs (*Canis lupus familiaris*) [117]. Weaknesses in biosecurity protocols, particularly in small pig farms, can facilitate disease transmission from wild boar populations. African swine fever, because of its virulence and the current epidemiologic situation in continental Europe, is one of the most relevant concerns in this regard [118]. Some waste management practices, such as offal incineration [119], safe disposal in carcass containers [116] (Jiménez et al., 2002), or avian scavengers [120], are considered effective strategies to control disease dispersal. Only 7.9% had access to carcass containers (normally associated with farm activity). Although Spain hosts the most important vulture breeding populations of all European countries [121], only 4.4% of collaborators use vulture feeding stations as places to leave the remains of game dressing. Vultures may contribute to the removal of ungulate infectious diseases due to their resistance to most pathogenic microorganisms [120]. Although being very effective in reducing biological contamination, incineration (0.34%) can be considered anecdotal. About wastewater, only 7.6% of the hunters interviewed declared having access to a specific organic residue water treatment plant. Most of the cases (53.5%) used the collective water network, and 30.2% released the polluted water to bodies of surface water or farmlands. Water has been proved to be an effective vehicle for animal–human shared pathogens such as *Mycobacterium* spp. [122,123,124] *Toxoplasma gondii* [125], or hepatitis E virus [126,127]. Uncontrolled wastewater coming from slaughterhouses can pollute water bodies with a plethora of parasites [128].

## 5. Conclusions

Public health authorities should increase their interest in topics regarding the self-consumption of game meat. There is an imperative need to provide formal training in good hygiene practices during all the steps of game meat preparation and handling. Specific cleaning protocols must be established, especially regarding the avoidance of cross-contaminations. Carcass containers must be available for game animal by-products to prevent environmental contamination and disease dissemination. Clear guidelines about domestic dressing facilities must be published to allow the users to improve their own facilities.

Specific health monitory programs should be implemented to detect wildlife diseases in the collective of hunters [129,130] to provide a quick response before any contagion and spreading to other people. It is essential to look for synergies with hunters’ associations, federations, and other stakeholders to promote a proactive approach. Additionally, it could be relevant to reevaluate the situation in the COVID-19 post-pandemic context due to the general use of personal protective equipment and disinfectants and a potential improvement of awareness and self-protection measures.

## Data Availability

Data sharing not applicable.

## Appendix A

**Questionnaire Model**
*(Score and prompts to future researchers are indicated in italics; these prompts must be removed in order to provide a raw questionnaire to future participants).*
**Hunting activity and game meat consumption survey**
The goal of this survey is to evaluate the factors related to hunting activities, the groups that practice it, the main target species, and habits about game meat handling and use. By completing it, you voluntarily agree to transfer the information, which will be used for research and scientific dissemination. Although you will be asked for some personal data, it is an anonymous survey, so you should not provide any type of information that allows your individual identification, sticking to the questions that are posed in the survey. That is why we ask that you should not provide any unsolicited information (such as your name, ID, or signature) in this document. Since the data requested are dissociated, that is, the information obtained cannot be associated with a specific or determinable person and no type of file will be generated that contains data that allow the identification of the participants, the application of the Law on Data Protection will not take place in the present case.
The organizing entities of this study thank you for your collaboration and invite you to consult the results once they are published.

I have read and understand the conditions of participation in this survey	
(mark with an X)	
Date **_____________________**	

**Block I. Generalities of the Game activity**

**1.- Gender**

Man		Woman	

**2.- Age**

18–30		31–50		51–65		>65	

**3.- Level of education**

No studies	
Primary studies	
Secondary–Professional studies	
University studies	

**4.- Work activity: ___________________________**

**  4.1.- Do you have any relation (employment/familiar) with livestock management?**

YES		NO	

**  4.2.- If affirmative, what kind of relation?** **_________________________________** **___ ___________________________________________________________________________________**

**5** **.- Place of residence: __________________________________________________________**

**6.- Municipalities where you mainly practice hunting**

  (a) Municipality 1 ______________________________ Province _______________

  (b) Municipality 2 ______________________________ Province _______________

  (c) Municipality 3 ______________________________ Province _______________

**7.- Hunting modalities & prey**

Driven hunting/Battue		Stalking		Stand hunting	
Wing shooting without dogs		Small game hunting with dogs		Calling decoy	
Ferret		Falconry		Dog without weapon	

Wild boar		Rabbit		Woodcock	
Red deer		Hare		Pheasant	
Fallow deer		Red-legged partridge		Duck	
Roe deer		Quail		Fox	
Corsican mouflon		Starling		Magpie	
Spanish wild ibex		Wood pigeon		Turtle dove	
Barbary sheep		Others (*which ones?*)			

**Block II. Game meat intake**

**8.- Do you regularly eat the meat of the animals you hunt?**

YES		NO	

**  8.1.- If not, why?**

Game meat is disgusting/repulsive	
Preparing game meat is too laborious	
You have eaten game meat for so long that it is boring nowadays	

**  8.2.- If you consume the meat**

**   8.2.1.- What species do you consume?** (Ordered by weight)

1.-	
2.-	
3.-	

**   8.2.2.- What is the destination of the meat?** (If more than one situation is possible, then indicate 1st, 2nd, and 3rd by relevance).

Self-consumption and inner circle	
Given to friends, neighbors, acquaintances…	
Selling	

**   8.2.3.- Do you freeze game meat before consumption?**

No, never		*0*				
Not normally		*0*				
Usually		*1*	**→**	Less than 1 month		*1*
Always		*2*		Between 1 and 2 m.		*2*
				Between 2 and 6 m.		*3*
				More than 6 months		*4*

**   8.2.4.- How do you gastronomically prepare game meat for consumption?**

Cooked		*3*
Raw-dried sausages		*0*
Salt-cured		*0*
Smoked		*1*
Pâté		*2*
Pickled		*2*
Raw		*0*

**Block III. Hunted Animals Management**

**9.- Do you call for veterinary inspection after hunting activities?**

Always		*2*
Usually		*1*
Only for *Trichinella* diagnosis when hunting wild boar		*1*
Not normally		*0*
Never		*0*

**10.-Hunted prey transport**

**  10.1.- How are hunted animals transported?**

Inside a personal/private vehicle			*1*
In an exclusive vehicle or trailer with a refrigeration system			*2*
In the back of a pickup with canopy or enclosed trailer			*1*
In the back of an open pickup, open trailer, or on the roof of a trailer			*0*
Others (specify):			*DA **
* *The score depends on the answer.*			

**  10.2.- Do you have authorization to transport ABP not intended for human consumption?** (Animal by-Products)

YES		*1*
NO		*0*

**  10.3.- How long does it normally take from the death of the animal until dressing?**

The dressing is done immediately in the field			*3*
Takes less than 2 h.			*2*
Takes between 2 and 4 h.			*1*
Takes between 4 and 6 h.			*0*
Takes between 6 and 8 h.			*0*
Takes more than 8 h			*0*
Others (specify):			*DA**
* *The score depends on the answer.*			

**11.- About personal hygiene and health protection while dressing**

**  11.1.- Do you wear any specific clothing to butcher?**

No, I use the same clothes as in the field			*0*
Yes, I change all the clothes to use a specific clothing			*2*
I use covering clothes (such as a laboratory coat or a coverall)			*1*
I use specific footwear (such as rubber boots)			*1*
Others (specify):			*DA **
* *The score depends on the answer.*			

**  11.2.-****Do you use personal protective equipment (against cuts, pricks, inhalations…) during dressing** (Several options can be marked if needed).

Disposable gloves			*1*
Leather gloves			*1*
Steel mesh gloves			*1*
Facial mask			*1*
Googles or face shield			*1*
Others (specify):			*DA **
I do not use any protective equipment			*0*
* *The score depends on the answer.*			

**  11.3** **.- The cutting tools (knives, kitchen scissors…) you use, are they specific for dressing game animals?**

Yes, they are exclusive for game meat		*1*
No, the tools are used in other tasks too		*0*

**  11.4.-****Can you wash your hands while handling carcasses and viscera?** (Several options can be marked if needed).

No, I cannot wash my hands immediately			*0*
Yes, it is running water			*2*
Yes, water coming from a water well, rainwater, etc.			*1*
Yes, I use soap			*2*
Yes, I use hand sanitizers			*2*
Others (specify):			*DA **
* *The score depends on the answer.*			

**12.- Ownership of the dressing site:**

Private own facilities		
Private facilities, owned by a different person		
Shared collective property (hunting club or similar)		
Public facilities, granted to hunters		
Others (specify):		
		

**13.- Dressing facilities description: resources and infrastructures.**

**  13.1.- Location**

Outdoors		*0*
Indoors		*1*

**  13.2.- Do you use the room for other purposes besides game dressing?** (Several options can be marked if needed).

No, it is intended exclusively for the **dressing** of hunted game			*2*
Game **dressing** and pantry			*0*
Game **dressing** and storage room			*1*
Game **dressing** and garage			*1*
Game **dressing** and workshop			*1*
Game **dressing** and meeting place			*1*
Game **dressing** and yard			*0*
Game **dressing** and others (specify):			*DA **
* *The score depends on the answer.*			

**  13.3.- Where do you place the animals to slaughter them?**

On the ground, on a dirt floor			*0*
On the ground, over a plastic sheeting			*1*
On the ground, on a pavement floor			*2*
On a table or bench			*3*
Hanging them			*4*
Others (specify):			*DA **
* *The score depends on the answer.*			

**  13.4.- Do you have water in the facility?**

Yes, hot and cold drinking water		*3*
Yes, cold drinking water		*2*
Yes, but it is not running water (rainwater, water well)		*1*
No		*0*

**  13.5.- What is the light source in the facility?**

Sunlight			*1*
Portable manual devices such as torches and headlamps			*2*
Lighting provided by a portable electric generator			*3*
Lighting provided by power grid and incandescent bulbs			*4*
Lighting provided by power grid and halogen tubes			*4*
Lighting provided by power grid and LED lights			*4*
Others (specify):			*DA **
* *The score depends on the answer.*			

**  13.6.- Pest control** (Several options can be marked if needed).

No control measures against insects			*0*
No control measures against rodents			*0*
Insect gauzes			*1*
Insect light traps			*1*
Insect adhesive traps			*1*
Insecticide sprays			*1*
Mouse spring trap			*1*
Rodent cage traps			*1*
Mouse glue traps			*1*
Rat poison			*1*
Others (specify):			*DA **
* *The score depends on the answer.*			

**  13.7.- How do you clean the facility after using it?** (Several options can be marked if needed).

I do not clean the facility			*0*
Hosing down			*1*
Mopping the floor			*1*
Dry brushing the floor			*0*
Scouring pads for specific surfaces			*1*
Use of bleach			*2*
Use of caustic soda			*1*
Use of ammonia			*1*
Use of floor cleaners			*1*
Use of soap			*1*
Others (specify):			*DA **
* *The score depends on the answer.*			

**  13.8.- Wastewater elimination.**

Through a specific organic wastewater treatment plant			*2*
Through the water network			*1*
To the outdoors (rivers, ravines, or crop fields)			*0*
Unknown			*0*
Others (specify):			*DA **
* *The score depends on the answer.*			

**  13.9.- Where are the butchered remains not intended for human consumption disposed of?** (Several options can be marked if needed).

Directly in the field, in an open area			*0*
Directly in the field, hidden in a pit cave, gully…			*0*
Directly in the field, in a burial plot			*1*
Vulture feeding station			*1*
Garbage containers			*1*
Carcass containers			*2*
Domestic dogs feeding			*0*
Others (specify):			*DA **
* *The score depends on the answer.*			
